# Severity-dependent and sex-biased dysregulation of A-to-I RNA editing in COVID-19 reveals CTSS as a key regulatory target

**DOI:** 10.1080/15476286.2026.2736869

**Published:** 2026-10-14

**Authors:** Jing-Yuan Wang, Ya-Di Zhao, Li-Ping Wang, Yu-Qing Ren, Jia-Nuo Li, Teng Wang, Hua-Kang Zhang, Feng Zhu, Jian-Huan Chen, Yun-Yun Jin

**Affiliations:** a Laboratory of Genomic and Precision Medicine, Wuxi School of Medicine, Department of Respiratory and Critical Care Medicine, Affiliated Wuxi Fifth Hospital of Jiangnan University, Jiangnan University, Wuxi, Jiangsu, China; b Joint Primate Research Center for Chronic Diseases, Institute of Zoology of Guangdong Academy of Sciences, Jiangnan University, Wuxi, Jiangsu, China; c MOE Medical Basic Research Innovation Center for Gut Microbiota and Chronic Diseases, Wuxi School of Medicine, Jiangnan University, Wuxi, Jiangsu, China; d Department of Respiratory and Critical Care Medicine, Affiliated Wuxi Fifth Hospital of Jiangnan University, Wuxi, Jiangsu, China; e School of Food Science and Technology, Jiangnan University, Wuxi, Jiangsu, China

**Keywords:** SARS-CoV-2, A-to-I RNA editing, ADAR, *CTSS*, disease severity, sex bias

## Abstract

SARS-CoV-2 infection causes diverse clinical outcomes, with men facing higher risks of severe disease. Adenosine-to-inosine (A-to-I) RNA editing mediated by ADAR enzymes governs innate antiviral immunity, yet its contribution to COVID-19 severity and sex-biased disease remains unclear. We performed epitranscriptomic profiling on 126 blood samples from non-COVID controls and COVID-19 patients spanning different disease severities. We detected 1,466 differentially RNA-edited sites in 514 genes linked to clinical outcomes. Although ADAR expression increased in COVID-19 patients overall, it was strongly reduced in ICU patients compared with non-ICU patients, and this shift was far more prominent in males. This severity-associated and sex-biased ADAR/ADARB1 downregulation coincided with decreased global RNA editing and perturbed expression of immune-related edited transcripts. Cathepsin S (CTSS) was identified as a central regulatory node: elevated A-to-I editing within its 3’ UTR in ICU patients stabilizes CTSS mRNA. Without stratifying by disease severity, ADAR expression and global editing showed no significant sex differences; the large sex divergence only appeared among ICU patients. This indicates severity-dependent, not infection-innate, disruption of A-to-I editing, disproportionately amplified in critically ill males. CTSS 3’UTR editing may serve as a biomarker for patient stratification, revealing a mechanistic connection between epitranscriptomic changes and male-biased severe COVID-19.

## GRAPHICAL ABSTRACT

**Figure:**
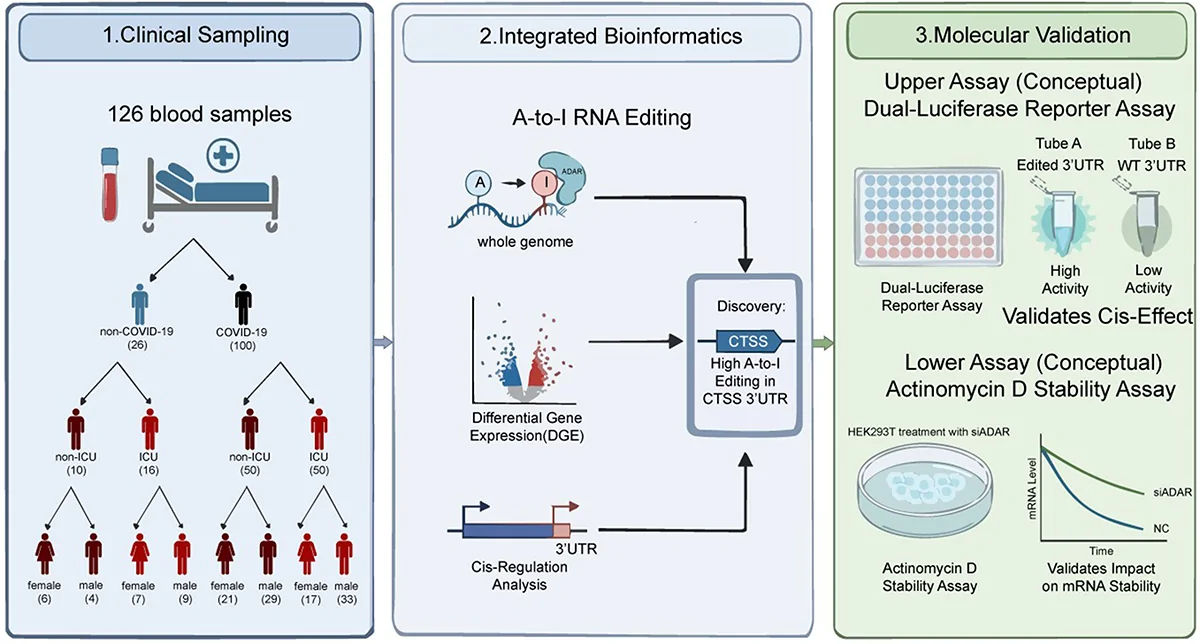

## Introduction

The emergence of SARS-CoV-2 in late 2019 precipitated a global COVID-19 pandemic, constituting a profound public health and societal crisis [1]. The clinical presentation of the disease is markedly heterogeneous, encompassing a spectrum from asymptomatic carriage to severe manifestations including acute respiratory distress syndrome (ARDS) and multi-organ failure [2,3]. Notably, epidemiological analyses have consistently indicated a sex disparity in outcomes, with male patients exhibiting disproportionately higher risks of severe morbidity and mortality compared to females [4,5]. This pattern has been corroborated by international surveillance data, establishing male sex as an independent risk factor for adverse clinical trajectories following SARS-CoV-2 infection [6–8]. The pronounced variability in disease severity, particularly between the sexes, poses a substantial challenge for clinical management and prognostic assessment, yet the fundamental molecular mechanisms underlying these disparities remain poorly elucidated.

Epigenetic mechanisms, particularly adenosine-to-inosine (A-to-I) RNA editing, serve as key modulators of transcriptomic complexity and function [9,10] and are critically involved in regulating innate antiviral immunity [11,12] and viral replication cycles [13,14]. This post-transcriptional modification, catalysed by adenosine deaminase acting on RNA (ADAR) enzymes [13], introduces specific sequence alterations in RNA molecules [15]. Such edits can influence multiple aspects of RNA metabolism, including splicing, stability, and translation, thereby potentially altering the function of the encoded protein [16]. Notably, evidence indicates that the expression of ADAR enzymes and associated immune pathways may be regulated by sex hormone signalling [17–20], positioning them as potential mediators of sex-specific biological effects. Furthermore, existing studies suggest that SARS-CoV-2 infection dynamically alters global patterns of A-to-I editing [21], a perturbation hypothesized to subsequently affect both disease severity and host immune responses in COVID-19 [22]. These observations lead us to propose that A-to-I RNA editing constitutes a significant yet underexplored intrinsic regulatory layer that shapes the immune and inflammatory landscape during infection and may influence clinical outcomes. Consequently, severity is a crucial variable that may shape distinct RNA editing landscapes, providing vital context for investigating the molecular determinants of gender in COVID-19.

In summary, this study’s findings highlight a significant relationship between A-to-I RNA editing dynamics in COVID-19 and both patient clinical severity and gender. By conducting transcriptome-wide profiling of blood-derived RNA editing patterns across a diverse cohort with various infection outcomes, this work improves our understanding of the role of epitranscriptomic regulation in the host’s antiviral response and identifies potential biomarkers for disease stratification.

## Materials and methods

### RNA-Seq reads download and processing

To examine alterations in RNA editing associated with COVID-19, raw RNA-Seq reads were obtained from the European Nucleotide Archive (ENA; accession PRJNA660067) (https://www.ebi.ac.uk/ena). This dataset comprises peripheral blood samples and corresponding clinical metadata from 126 adult individuals, including 100 confirmed SARS-CoV-2-positive patients and 26 negative controls, as previously described [23]. The cohort includes both male and female participants across a spectrum of disease severity. ICU and non‑ICU subgroups were defined using two composite severity metrics: hospital‑free days at day 45 (HFD‑45) and the WHO ordinal scale at day 28. HFD‑45 assigns a value of 0 to patients who died or remained hospitalized beyond 45 days, reflecting the most severe disease burden, whereas shorter hospital stays yield higher scores; the WHO scale (0–8) similarly captures clinical severity and mortality [23]. Using these measures, the 100 COVID‑19‑positive patients were stratified into ICU (*n* = 50) and non‑ICU (*n* = 50) subgroups, while the 26 negative controls were classified into ICU (*n* = 16) and non‑ICU (*n* = 10) subgroups. Meanwhile, the PRJNA614963 dataset, which involved ADAR knockdown using siRNA in A549 cells, was used to analyse ADAR’s regulatory role in RNA editing [24]. A549 cells, a human lung adenocarcinoma epithelial cell line, are a widely used model for respiratory virus research, including COVID-19.

Data processing followed an established analytical workflow. Initial sequence quality assessment was performed using FASTQC [25]. High-quality reads were then aligned to the human reference genome (UCSC hg38) with RNA STAR (v2.7.0e) [26]. Subsequent filtering of the resulting BAM files was conducted with SAMtools (v1.9) to remove reads with multiple mapping positions and PCR duplicates [26]. Finally, base quality score recalibration was applied using the Genome Analysis Toolkit (GATK v4.1.3) [27].

### RNA editing analysis

Putative single-nucleotide variants (SNVs) were called from the processed BAM files using VarScan (v2.4.3) [28]. Variant annotation was subsequently performed with the Ensembl Variant Effect Predictor (VEP) [29]. To isolate high-confidence editing candidates, a multi-step filtration strategy was applied. Initial candidates were required to meet the following quality thresholds: a minimum base quality score of 25, a total read depth of at least 10, an alternative allele depth of at least 2, and an alternative allele frequency (AAF) of at least 1%.

To exclude the possibility that observed differences in editing site counts between groups were driven by sequencing depth, we first compared the total number of mapped reads per sample across clinical groups. No significant differences were detected between COVID‑19 patients and non‑COVID controls (*p* > 0.05, Wilcoxon rank‑sum test), nor between ICU and non‑ICU groups, indicating that the subsequent editing analyses were not confounded by variations in overall coverage.

To facilitate cross‑sample comparisons of editing frequencies, we normalized the allele fraction at each candidate site. Briefly, the raw alternative allele count was divided by the total read depth to obtain the unadjusted editing ratio. To mitigate technical variability arising from library preparation and sequencing depth, we applied median‑centring normalization within each site, using an empirical Bayes shrinkage method implemented in the R package REDITs. This procedure subtracts the site‑specific median from each sample’s ratio and then adds the global median, thus reducing batch effects while retaining biological differences. Normalized values outside the [0,1] interval were winsorized to the boundaries of the interval. Previous evaluations have shown that this approach improves the sensitivity of differential editing detection and reduces false positives in RNA‑seq datasets.

Further stringent filters were then employed to exclude artefacts and potential genomic polymorphisms. Candidates were removed if they met any of the following criteria, except for sites previously documented in the REDIportal V2.0 database of known RNA editing events [30]: 1) location within homopolymer runs of five or more nucleotides or in simple repeats; 2) situated in mitochondrial DNA; 3) proximity within 6 nucleotides of an annotated splice junction; 4) within 1 nucleotide of an insertion-deletion (INDEL); 5) within the first or last 4% of aligned reads; 6) annotation as a known single nucleotide polymorphism in dbSNP (database Build 142; 7) more than 90% of samples had an allele frequency of 100% or between 40% and 60%, with an editing level below 5% across all COVID-19 groups.

To characterize the nucleotide composition of the identified editing sites, we calculated the distribution of all 12 possible mismatch types (A > G, A > C, A > T, G > A, G > C, G > T, C > A, C > G, C > T, *T* > A, *T* > C, *T* > G) among the high‑confidence candidates. Of the 56,429 filtered variants, A‑to‑G transitions accounted for 27,992 sites (49.62%), far exceeding any other mismatch category. This marked predominance of A‑to‑G substitutions – the canonical signature of ADAR‑mediated A‑to‑I RNA editing – strongly supports the specificity of our calling pipeline and confirms that the vast majority of identified events arise from adenosine deamination rather than from sequencing artefacts or other mutation types.

### Sorting intolerant from tolerant (*SIFT*) prediction

The *SIFT* online platform (http://sift-dna.org) was used to predict the effects of amino acid mutations in proteins resulting from 3’ UTR A-to-I RNA editing [31].

### Gene expression quantification

The Rsubread package in R was used to compute pseudo-counts of RNA expression and to normalize gene expression levels to transcripts per million (TPM) [32–34].

### Function enrichment analysis

The DAVID online platform (https://david.ncifcrf.gov/tools.jsp) and Enrichr (https://maayanlab.cloud/Enrichr/) were used to conduct Gene Ontology (GO) and Kyoto Encyclopaedia of Genes and Genomes (KEGG) analyses [35]. Items with a significance level below 0.05 were deemed significant based on the false discovery rate (FDR).

### Knockdown of ADAR using siRNA in HEK293T cells

Chemically synthesized siRNA duplexes targeting human *ADAR* (NM_001111.5) and a negative‑control siRNA lacking sequence homology to the human genome were obtained from a commercial supplier (sequences provided in Table S1). Human embryonic Kidney 293T cells were grown in DMEM supplemented with 10% fetal bovine serum and penicillin-streptomycin at 37°C under 5% CO_2_. Cells were transfected with siRNA using Lipomaster 3000 Transfection Reagent following the manufacturer’s instructions and subjected to subsequent RNA extraction.

### DNA and RNA extraction

Genomic DNA and total RNA were extracted from the collected cell samples. DNA was extracted using the FastPure Cell/Tissue DNA Isolation Mini Kit (Vazyme, Cat: DC102-01) according to the manufacturer’s instructions. Total RNA was extracted using the RNAsimple Total RNA Kit (Tiangen, REF: DP419) according to the manufacturer’s instructions. The concentration and quality of nucleic acids were measured using a NanoDrop One spectrophotometer (NanoDrop, Thermo Fisher Scientific), and RNA integrity was further assessed by agarose gel electrophoresis.

Reverse transcription of RNA was performed using the HiScript III RT SuperMix for qPCR (+gDNA wiper) (Vazyme, R323) according to the kit protocol for downstream analyses [36].

### PCR amplification for RNA editing site validation

To verify RNA editing at specific loci, primers flanking the target sites were designed (Table S1). PCR amplification was performed using genomic DNA and cDNA as templates with the corresponding locus-specific primers. PCR was performed using Phanta Max Super-Fidelity DNA Polymerase (Vazyme, P505) according to the manufacturer’s protocol. After analysis by agarose gel electrophoresis, PCR products were directly subjected to Sanger sequencing to identify the precise positions and types of RNA editing events, including A-to-I editing at the target sites.

### Plasmid construction and dual-luciferase reporter assays

To investigate the functional impact of A-to-I RNA editing within the 3’ untranslated regions (UTRs), dual-luciferase reporter assays were performed for the *CTSS* loci. Genomic fragments encompassing either the wild-type (WT) sequence or the edited variant (ET), where the target adenosine was substituted with guanosine to mimic inosine, were synthesized. These fragments were cloned into a psiCHECK-2 vector, downstream of the firefly luciferase gene. The integrity of all plasmid constructs was confirmed by Sanger sequencing prior to experimental use.

HEK293T cells were seeded in 24-well plates and transfected when they reached 70–80% confluency [37]. Cells were co-transfected with either the WT or ET reporter plasmid and a *Renilla luciferase* control plasmid using Lipofectamine 3000 (Thermo Fisher Scientific) according to the manufacturer’s instructions. Firefly and *Renilla luciferase* activities were measured 48 hours post-transfection using the Dual-Luciferase Reporter Assay System (Promega). Firefly luciferase activity was normalized to the internal *Renilla luciferase* control to correct for transfection efficiency. Relative luciferase activity was then compared between WT and ET constructs.

### Quantitative real-time PCR (qRT-PCR)

We performed qRT-PCR using SYBR Green Master Mix. Primer sequences for *CTSS*, *ADAR*, and beta-actin (*ACTB*, the internal reference gene) are listed in Table S1. Relative mRNA expression levels were calculated using the comparative Ct (2^–ΔΔCt^) method [38]. All reactions were performed in technical triplicate, and the experiments were biologically replicated at least three times [39].

### RNA-binding protein (RBP) binding site prediction

For a better understanding of the potential functional impact of RNA editing, the RBPmap online tool (http://rbpmap.technion.ac.il) was employed to predict potential RBP binding sites that coincide with the locations of RNA editing [40].

### Actinomycin D-Based mRNA stability assay

To evaluate the impact of *CTSS* editing on mRNA stability, an actinomycin D (Act D) assay was performed. HEK293T cells were transfected with either the WT or ET *CTSS* 3’ UTR reporter plasmid. Six hours post-transfection, the culture medium was replaced with fresh medium, and then 5 µg/mL actinomycin D (Sigma-Aldrich, A9415) was added to globally inhibit transcription at different time points [41]. Control wells received an equivalent volume of DMSO vehicle. Total RNA was harvested at defined intervals post-Act D addition (0, 4, and 8 hours) from triplicate wells. The mRNA half-life of *CTSS* was then determined by qRT-PCR using primers listed in Table S1. Values were normalized to the 0-hour time point to calculate the percentage of mRNA remaining, and decay curves were plotted to compare the stability of transcripts containing the WT versus ET 3’ UTR.

### Statistical analysis

Generalized linear models (GLMs) were used to assess differences in RNA editing levels among the COVID‑19 clinical groups. For each editing site, the response variable was defined as the number of alternative reads relative to total read depth per sample. The model included clinical group as a fixed factor, with sex and severity included as covariates where appropriate. Empirical *P*‑values (*P*_GLM_) were derived from likelihood ratio tests comparing the full model against a reduced model without the group term. The GLM tests were supplemented by *Fisher*‘s exact test for further evaluation using allele read depths. For comparisons of individual RNA editing sites and genes between groups, *Student*’*s* t-test was applied. Associations between continuous variables were evaluated using *Spearman*‘s rank correlation coefficient. The correlation coefficient (*r*) and its associated *P*‑values are reported for each tested relationship.

## Result

### A-to-I RNA editing was identified in the samples of 8 groups

Characteristics of the 126 samples, including sex, disease status, and ICU admission status, are summarized in Table 1. In all recipients’ blood, a total of 27,992 high-confidence A-to-I RNA editing sites were identified in 2,769 genes (Figure 1(A)). Over the whole human genome, the editing levels of these events ranged from 1% to 100%. The three most prevalent functional categories of these RNA editing events were: 13,515 (48.3%) 3’ untranslated region (3’ UTR), 9,898 (35.4%) intronic, and 2,137 (7.6%) missense variants (Figure 1(B)). *SIFT* analysis suggested that 924 (43.3%) of the missense variants could potentially affect the function of the encoded proteins (Figure 1(C)). 54.2% of these edited sites coincided with *Alu* repetitive elements (Figure 1(D)). Circos plotting of ADAR‑editing correlations revealed that multiple DRE sites across the genome showed significant positive correlations with *ADAR* expression (*r* > 0.3, *p* < 0.05) (Figure 1(E)), suggesting ADAR‑dependent regulation across the transcriptome.

**Figure 1.**
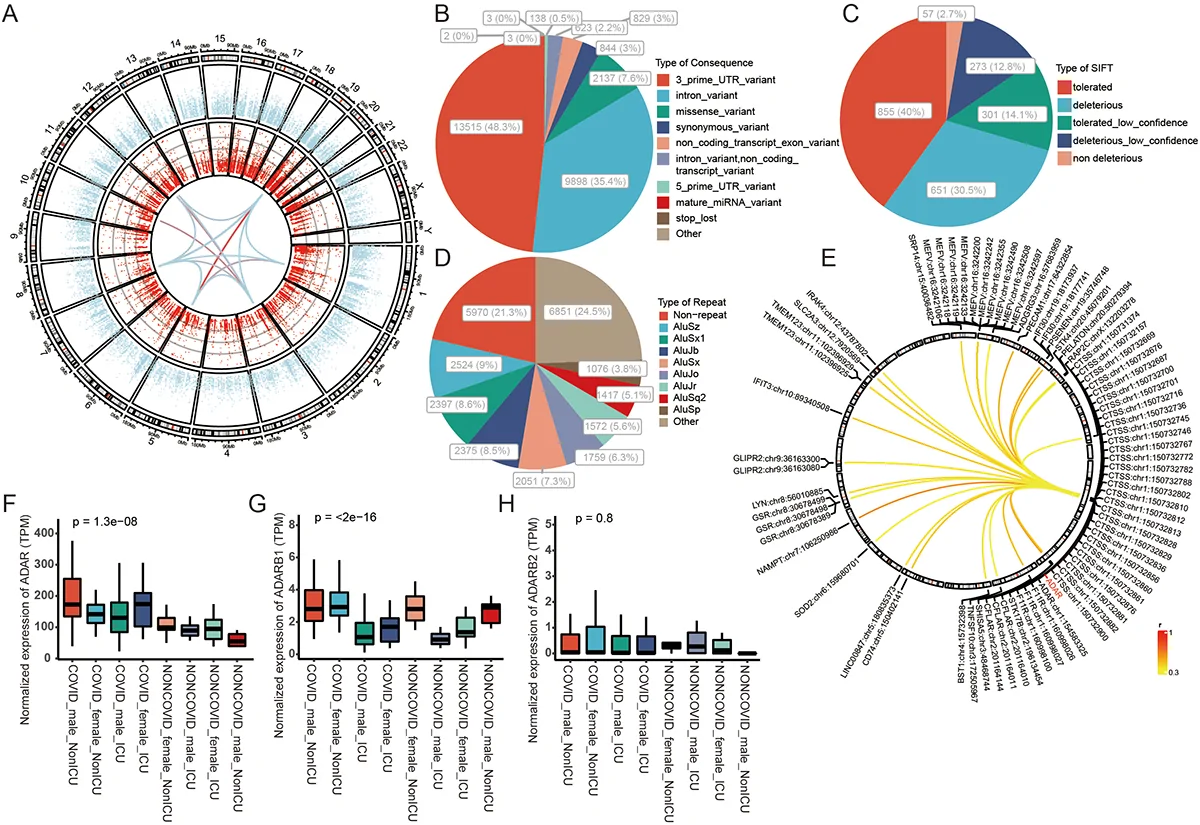
A-to-I RNA editing was identified from the blood transcriptome of the COVID-19 cohort. (A) Circos plot of transcription gene expression (the outer circle) and A-to-I RNA editing sites (the inner circle) in 126 human blood samples. (B) Functional variants resulting from A-to-I RNA editing. (C) About 43.3% of missense variants are predicted by *SIFT* to be possibly deleterious to the encoded proteins. (D) 54.2% of RNA editing events occur within Alu repeat elements. (E) The correlation between *ADAR* and the editing levels of individual DRE sites in the cohort (*n* = 126). Each point represents a DRE site at its genomic coordinate. The radial distance of each point represents the *Spearman* correlation coefficient (*r*) between *ADAR* expression levels (TPM) and A‑to‑I editing levels. The color scale (or point size) further indicates the significance level. (*Spearman* correlation *r* > 0.3 and *p* < 0.05). (F–H) the expression of *ADAR* (F), *ADARB1* (G), and *ADARB2* (H) in eight groups stratified by COVID-19 status, ICU care, and sex (*N* = 30 for COVID-19 non-ICU males; *N* = 21 for COVID-19 non-ICU females; *N* = 34 for COVID-19 ICU males; *N* = 17 for COVID-19 ICU females; *N* = 6 for non-COVID-19 non-ICU females; *N* = 9 for non-COVID-19 ICU males; *N* = 7 for non-COVID-19 ICU females; *N* = 4 for non-COVID-19 non-ICU males). Overall and post hoc *p*-values are calculated using the ANOVA and Tukey tests. We calculated the overall *p*-value using the GLM model and considered *p* < 0.05 statistically significant. GLM: generalized linear model.

**Table 1.** The demographic characteristics of the study subjects.

Group		Male	Female	Total
COVID-19	non-ICU	29	21	50
	ICU	33	17	50
non-COVID-19	non-ICU	4	6	10
	ICU	9	7	16
Total		75	51	126

Across the eight transcriptomic subgroups defined by infection status, ICU requirement, and sex, the expression levels of *ADAR* and *ADARB1* showed significant variation, as determined by generalized linear models (*P*_GLM_ < 0.05; Figure 1(F, G)), whereas *ADARB2* did not exhibit significant differences (Figure 1(H)). Specifically, pairwise comparisons revealed that the most prominent differences for both *ADAR* and *ADARB1* were between COVID‑19 male non‑ICU patients and non‑COVID‑19 male non‑ICU individuals (*ADAR*: *p* = 1.3 × 10^−8^; *ADARB1*: *p* < 2 × 10^−16^). These findings indicate that *ADAR* and *ADARB1*, but not *ADARB2*, are likely key enzymatic drivers of the editing landscape in severe COVID‑19. Notably, sex stratification revealed opposing trends in *ADAR* expression between male and female patients across clinical groups, prompting us to further investigate sex‑specific editing patterns in subsequent analyses.

### RNA editing in response to COVID-19 in blood transcriptome

Compared with the non-COVID-19 cohort, the *ADAR* and *ADARB1* expression levels were substantially higher in COVID-19 (Figure 2(A,B)). Consistently, it was observed that the number of RNA editing genes and sites exhibited a significant increase in COVID-19 relative to non-COVID-19 (Figure 2(C,D)). *Spearman* correlation analysis showed that average RNA editing levels in COVID-19 and non-COVID-19 groups were positively correlated with *ADAR* and *ADARB1* expression (Figure 2(E,F)). Notably, the correlation with *ADAR* was consistently stronger than that with *ADARB1* in the cohort. Moreover, 24,215 (86.2%) of these RNA editing sites were commonly identified between non-COVID-19 and COVID-19. In contrast, 131 (0.5%) and 3739 (13.3%) were exclusively observed in the non-COVID-19 and COVID-19 groups, respectively (Figure 2(G)). Among edited genes, 2,520 (90.7%) were shared, with 9 (0.3%) and 249 (9.0%) genes observed in the non-COVID-19 and COVID-19 groups, respectively (Figure 2(H)).

**Figure 2.**
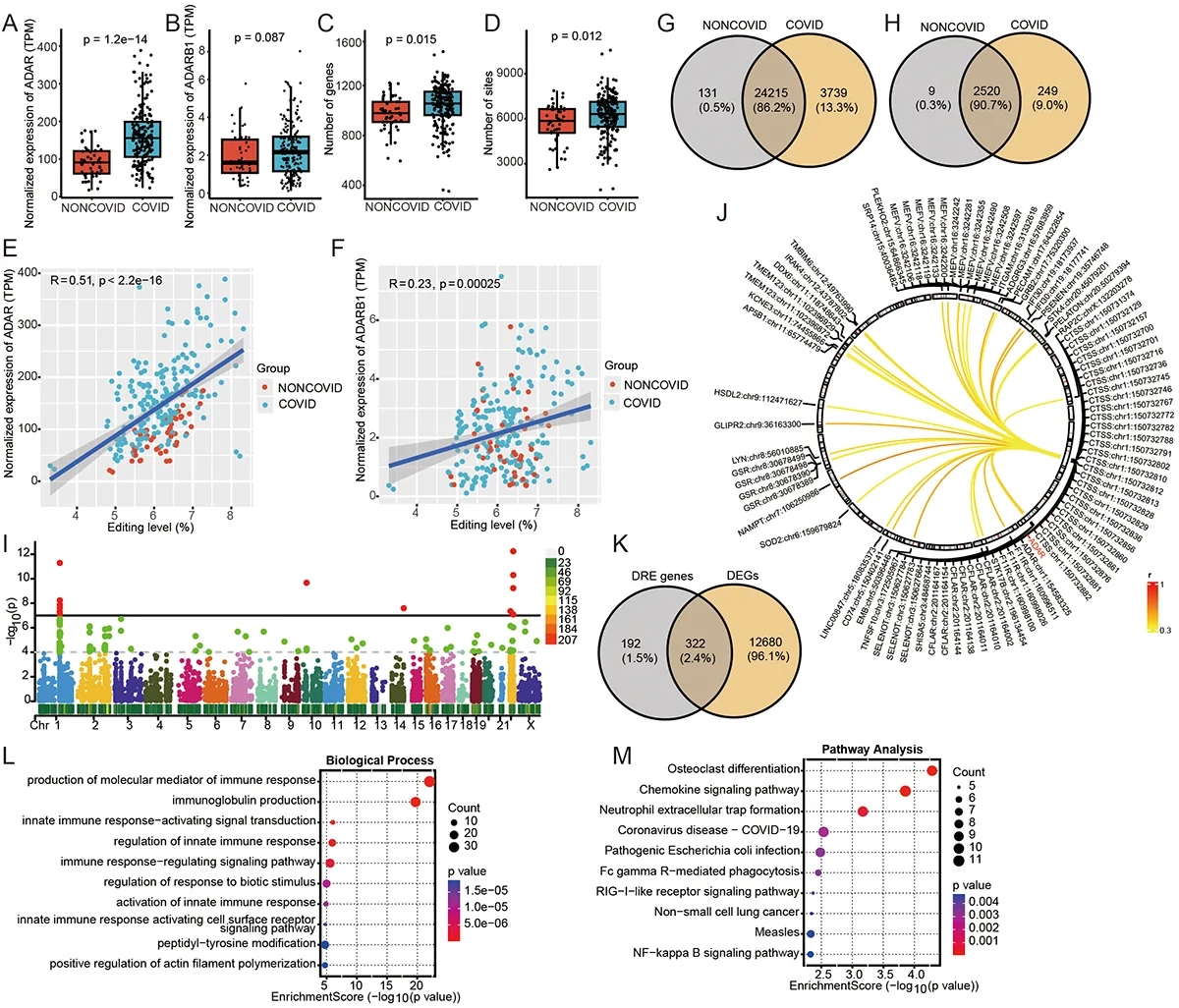
A-to-I RNA editing was identified in the blood transcriptome cohorts of COVID-19 and non-COVID-19. (A-B) The expression of *ADAR* and *ADARB1* in the COVID-19 and non-COVID-19 groups (*N* = 100 for COVID-19; *N* = 26 for non-COVID-19, respectively). (C-D) The number of edited genes and sites in the two groups. *p* < 0.05. Overall and post hoc *p*-values are calculated using the ANOVA and Tukey tests. (E-F) The *Pearson* correlation of the overall average editing level and gene expression of *ADAR* and *ADARB1* in samples. (G-H) The Venn plot showed the shared DRE sites (G) and genes (H) across different patient datasets. (I) Manhattan plot showing the distribution of all detected editing sites across the genome in COVID-19 patients versus non‑COVID controls. The heavy black horizontal line denotes the significance threshold of *p* = 1 × 10^−7^, while the dashed grey line indicates a nominal significance threshold of *p* = 1 × 10^−4^. All tested sites, irrespective of their significance levels, are plotted; the density plot below shows the density of editing sites using a sliding-window method. (J) Circos plot of the correlation between *ADAR* expression and editing levels of DRE sites between COVID-19 and non‑COVID‑19. *Spearman* correlation *r* > 0.3 and *p* < 0.05. (K) The Venn plot showed the shared genes between DRE genes and DEGs in COVID-19. (L-M) The items are shown for biological processes (L) and KEGG pathways (M) enriched by the 322 shared genes. *p*-values are calculated using the independent *Student’s* t-test. All tests were two-sided, and a *p*-value < 0.05 was considered statistically significant.

Notably, among these RNA editing events, 1466 DRE sites were observed in 514 genes (Figure 2(I)). When focusing on DRE sites that were differentially edited between COVID‑19 and non‑COVID‑19 groups, as shown in Figure 2(J), a subset of DRE sites whose editing levels correlate with *ADAR* expression specifically during SARS‑CoV‑2 infection, supporting a role for *ADAR* in shaping the disease‑associated editing landscape. Moreover, 322 (2.4%) genes were shared between DREs and differentially expressed genes (DEGs) (Figure 2(K)). Functional enrichment analysis of the shared genes also demonstrated the influence of A-to-I RNA editing alterations on biological functions. The most markedly enriched biological processes were shown in the production of molecular mediators of immune response, immunoglobulin production, and innate immune response-activating signal transduction (Figure 2(L)); KEGG pathways were mainly related to the chemokine signalling pathway, coronavirus disease-COVID-19, and the RIG-I-like receptor signalling pathway (Figure 2(M)).

Among the identified immune-related significant sites, Apolipoprotein B MRNA Editing Enzyme Catalytic Subunit 3C (*APOBEC3C*:chr22:39018907), MDM2 Proto-Oncogene (*MDM2*:chr12:68843778), and Fc Gamma Receptor IIIa (*FCGR3A*:chr1:161542063) were notable. Compared with non-COVID-19, both RNA editing levels and gene expression levels at these sites were significantly elevated in COVID-19 (Figure S1A-S1F), and site-specific editing was strongly correlated with gene expression (Figure S1G-S1I).

### A-to-I RNA editing in COVID-19 is not associated with sex-specific susceptibility

Comparison between COVID-19 females and COVID-19 males showed that ADAR gene expression levels did not differ significantly (Figure S2A). Furthermore, no significant differences were found in the number of RNA editing sites, the number of RNA editing genes, or RNA editing levels (Figure S2B-S2D). In the absence of consideration for disease severity, there was no significant difference in RNA editing enzyme expression levels, suggesting no sex-based difference in SARS-CoV-2 susceptibility.

### A-to-I RNA editing in COVID-19 is related to severity

Compared with COVID-19 non-ICU, significantly decreased expression levels of *ADAR* and *ADARB1* were observed in the COVID-19 ICU (Figure 3(A,B)). Meanwhile, the global average A‑to‑I RNA editing levels, the number of RNA editing genes, and the number of RNA editing sites were significantly decreased in the COVID-19 ICU relative to the COVID-19 non-ICU (Figure 3(C,E)). *Spearman* correlation analysis showed that RNA editing levels were positively associated with *ADAR* and *ADARB1* expression (Figure 3(F,G)), with a stronger correlation observed for *ADAR*.

**Figure 3.**
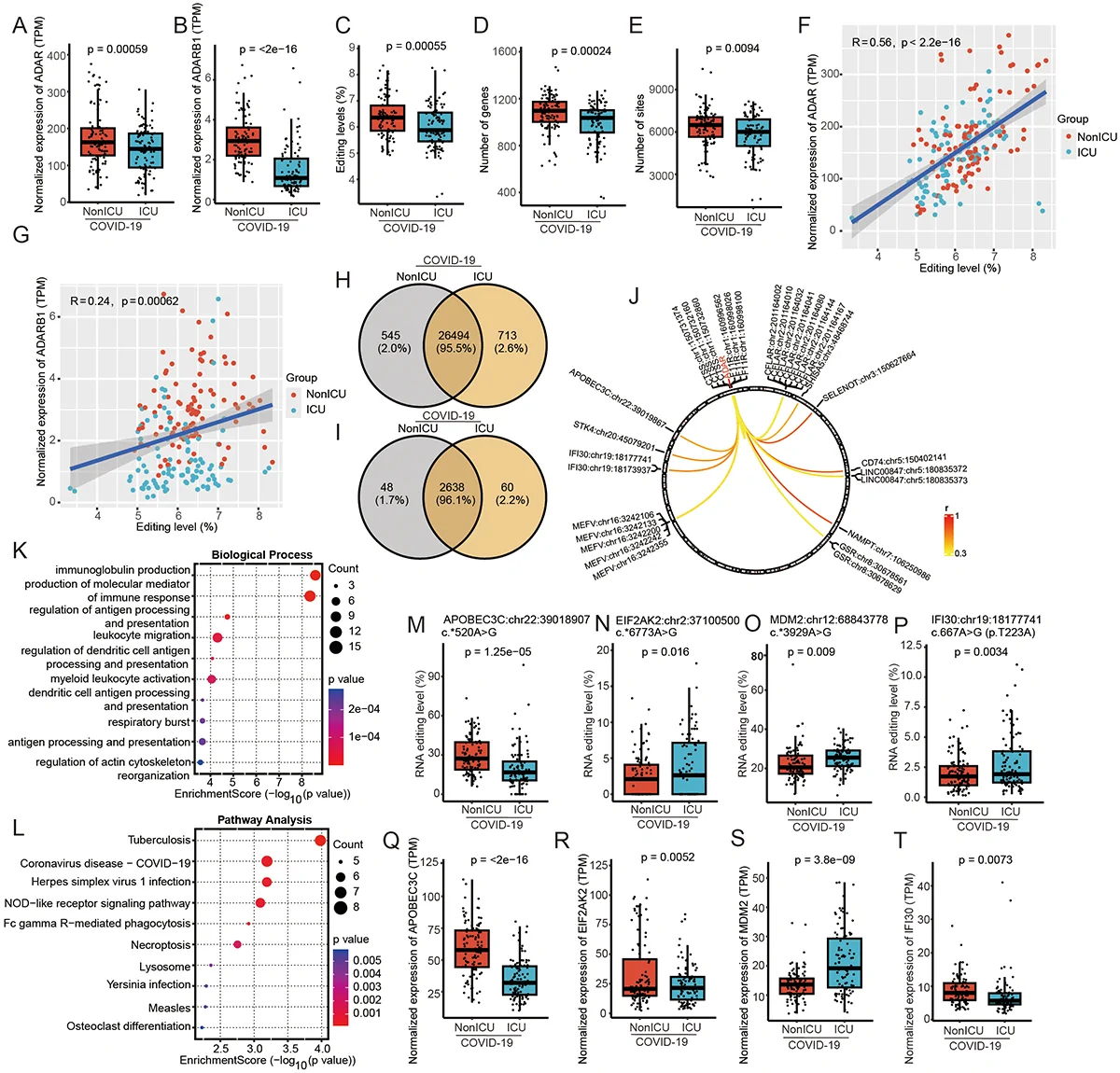
A-to-I RNA editing in ICU and non-ICU COVID-19 patients. (A-B) The mRNA expression of *ADAR* (A) and *ADARB1* (B) between the COVID-19 ICU and COVID-19 non-ICU (*N* = 50, respectively). (C) The average A‑to‑I RNA editing levels in COVID-19 ICU (*N* = 50) and COVID-19 non‑ICU (*N* = 50) patients. Each data point represents the editing level of an individual sample, expressed as a percentage. (D) Number of edited genes in ICU versus non‑ICU patients. (E) Number of editing sites in ICU versus non‑ICU patients. (F-G) *Spearman* correlation of the average editing level and gene expression of *ADAR* and *ADARB1* in two groups. (H-I) Venn plot showing A-to-I RNA editing sites (H) and genes (I) between COVID-19 non-ICU and COVID-19 ICU. (J) The correlation between *ADAR* and the editing levels of individual DRE sites in two groups. (K-L) The items are shown for biological processes (K) and pathways (L), enriched by genes differentially edited between non-ICU and ICU in COVID-19. (M-T) *APOBEC3C*:chr22:39018907, *EIF2AK2*:chr2:37100500, *MDM2*:chr12:68843778, and *IFI30*:chr19:18177741 A-to-I RNA editing level (M-P) and gene expression level (Q-T) between the COVID-19 ICU and COVID-19 non-ICU (*N* = 50, respectively). *p*-values were calculated using the independent *Student’s* t-test. All tests were two-sided, and a *p*-value < 0.05 was considered statistically significant.

Furthermore, shared RNA editing sites accounted for 26,494 (95.5%) of the total between the COVID-19 non-ICU and the COVID-19 ICU. In comparison, 545 (2.0%) and 713 (2.6%) editing sites were exclusively observed in COVID-19 non-ICU and COVID-19 ICU, respectively (Figure 3(H)). Regarding edited genes, 2,638 (96.1%) were shared between the COVID-19 non-ICU and the COVID-19 ICU, with 48 (1.7%) and 60 (2.2%) genes exclusively edited in COVID-19 non-ICU and COVID-19 ICU, respectively (Figure 3(I)). Further analysis showed that a subset of DRE events positively correlated with *ADAR* expression levels (Figure 3(J)). Of the 386 differential editing sites identified between COVID-19 ICU and COVID-19 non‑ICU patients, 121 were up‑regulated, and 265 were down‑regulated (Table S2). Gene function enrichment showed that the DRE observed between COVID-19 non-ICU and COVID-19 ICU was mainly involved in immune and virus-related biological processes, such as antigen processing and presentation, immunoglobulin production, and leukocyte migration (Figure 3(K)), and KEGG pathways, such as Coronavirus disease-COVID-19 and NOD-like receptor signalling pathway (Figure 3(L)).

Specifically, among the identified sites, Apolipoprotein B MRNA Editing Enzyme Catalytic Subunit 3C (*APOBEC3C*:chr22:39018907), Eukaryotic Translation Initiation Factor 2 Alpha Kinase 2 (*EIF2AK2*:chr2:37100500), MDM2 Proto-Oncogene (*MDM2*:chr12:68843778), and IFI30 Lysosomal Thiol Reductase (*IFI30*:chr19:18177741) were found to be significantly associated with immune responses and viral processes. In comparison with the COVID-19 non-ICU, all the editing levels were increased in the COVID-19 ICU except *APOBEC3C* (chr22:39018907) (Figure 3(M–P)). At the gene expression level, compared with the non‑ICU group, the ICU group exhibited decreased expression of *APOBEC3C*, *EIF2AK2*, and *IFI30*, while the expression of *MDM2* increased (Figure 3(Q-T)).

### A-to-I RNA editing is associated with sex in the COVID-19 ICU cohort

To assess sex‑specific differences in severe COVID‑19, we performed an analysis specifically focusing on the COVID‑19 ICU cohort. Significantly decreased levels of *ADAR* were observed in the COVID-19 ICU males compared with the COVID-19 ICU females, whereas *ADARB1* levels showed a downward trend (Figure 4(A,B)). *Spearman* correlation analysis further revealed that RNA editing levels were significantly correlated with *ADAR* and *ADARB1* expression (Figure 4(C,B)), and the association with *ADAR* was stronger than with *ADARB1*. Principal component analysis (PCA) using DRE sites effectively separates males and females in the ICU, with PC1 explaining 25.46% of the total variation (Figure 4(E)).

**Figure 4.**
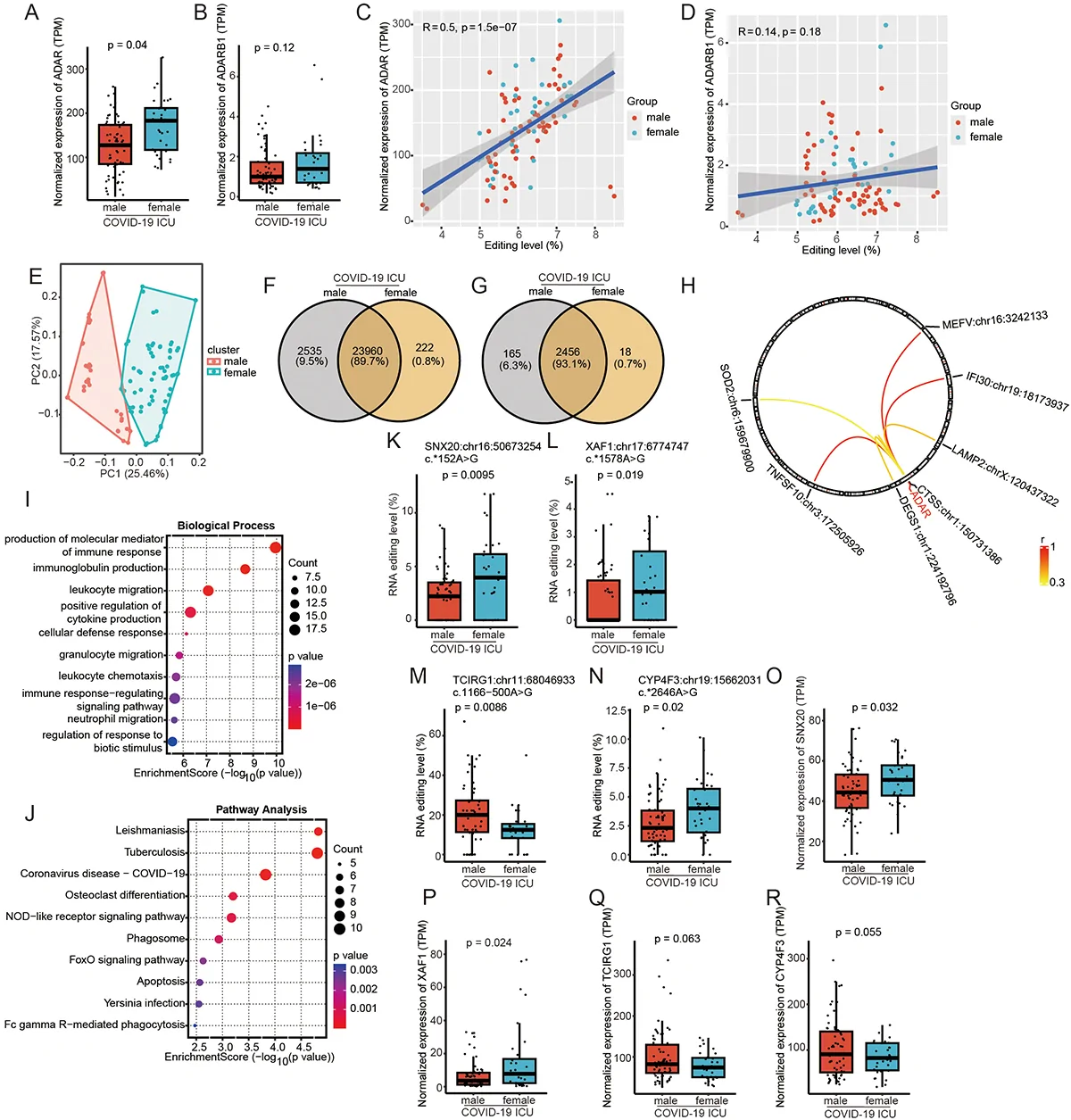
A-to-I RNA editing between males and females in the ICU. (A-B) The mRNA expression of *ADAR* (A) and *ADARB1* (B) between COVID-19 ICU males and females (*N* = 33 for ICU males; *N* = 17 for ICU females, respectively). (C-D) The *Spearman* correlation of average editing level and gene expression of *ADAR* (C) and *ADARB1*(D) in COVID-19 ICU males and ICU females. (E) PCA of DRE events between males and females in the ICU. (F-G) The Venn plot showed the shared DRE sites (F) and genes (G) in COVID-19 ICU males and females. (H) The correlation between *ADAR* and the editing levels of individual DRE sites in two groups. (I-J) The items are shown for biological processes (I) and pathways (J) enriched by differentially edited genes. (K-R) *SNX20*:chr16:50673254, *XAF1*:chr17:6774747, *TCIRG1*:chr11:68046933, and *CYP4F3*:chr19:15662031 A-to-I RNA editing level (K-N) and gene expression level (O-R) between the COVID-19 ICU males and females (*N* = 33 for ICU males; *N* = 17 for ICU females, respectively). *p*-values are calculated using the independent *Student’s* t-test. All tests were two-sided, and a *p*-value < 0.05 was considered statistically significant.

In these RNA editing sites, 23,960 (89.7%) were shared between the COVID-19 ICU male and the COVID-19 ICU female. In contrast, 2535 (9.5%) and 222 (0.8%) were exclusively shown in the ICU male and ICU female, respectively (Figure 4(F)). For edited genes, 2,456 (93.0%) were shared, with 165 (6.3%) and 18 (0.7%) genes observed in COVID-19 ICU males and COVID-19 ICU females, respectively (Figure 4(G)). A subset of these DRE sites was identified to exhibit a positive correlation with *ADAR* expression levels (Figure 4(H)). Functional enrichment analysis of biological processes and pathways was then performed on the DRE genes to elucidate the functional impact of A-to-I RNA editing changes. The DRE genes were mainly involved in biological processes related to immunoglobulin production, cellular defence response, and positive regulation of cytokine production (Figure 4(I)), and in pathways such as Coronavirus disease-COVID-19, NOD-like receptor signalling pathway, and Apoptosis (Figure 4(J)).

Among the DRE sites, those that are highly associated with immune and viral processes were identified, including Sorting Nexin 20 (*SNX20*:chr16:50673254), XIAP Associated Factor 1 (*XAF1*:chr17:6774747), T Cell Immune Regulator 1, ATPase H^+^ Transporting V0 Subunit A3 (*TCIRG1*:chr11:68046933), and Cytochrome P450 Family 4 Subfamily F Member 3 (*CYP4F3*:chr19:15662031). Compared with the COVID-19 ICU male, RNA editing levels were elevated in the COVID-19 ICU female for all sites with the exception of *TCIRG1* (chr11:68046933) (Figure 4(K-N)). Moreover, compared to ICU males, ICU females showed a significant increase in the expression levels of *SNX20* and *XAF1* (Figure 4(O,P)), while the expression of *TCIRG1* and *CYP4F3* was decreased (Figure 4(Q,R)).

### Sex-specific core DRE sites linking RNA editing to immune and viral responses in COVID-19 severity

Seven sites and 34 genes were commonly shared across the DREs between the COVID-19 ICU and non-ICU groups, DRE between the COVID-19 ICU males and females, and si*ADAR* (Figure 5(A,B)). Among the seven shared sites, the notable ones included Serine/Threonine Kinase 4 (*STK4*:chr20:45078299), F11 Receptor (*F11R*:chr1:160996561), and Cathepsin S (*CTSS*:chr1:150731222), which exhibited the strongest association with immune function and viral infection. Compared with the COVID-19 non-ICU, the RNA editing levels of the differential sites were universally elevated, with the exception of *F11R* (chr1:160996561) in the COVID-19 ICU (Figure 5(C,E,G)). Except for *F11R*, the expression levels of other genes were decreased in the COVID-19 ICU (Figure 5(D,F,H)). Additionally, compared with the ICU male, the RNA editing levels of all differential sites were significantly decreased in the ICU female (Figure 5(I,K,M)). Three gene expression levels have no significant change (Figure 5(J,L,N)). Furthermore, in the PRJNA614963 dataset, the RNA editing level of all DRE sites was substantially reduced in A549 cells treated with siADAR compared to the negative control (NC) (Figure 5(O,Q,S)). The expression, in the PRJNA614963 dataset, was increased in A549 cells treated with si*ADAR* compared with NC, except for *F11R* (Figure 5(P,R,T)).

**Figure 5.**
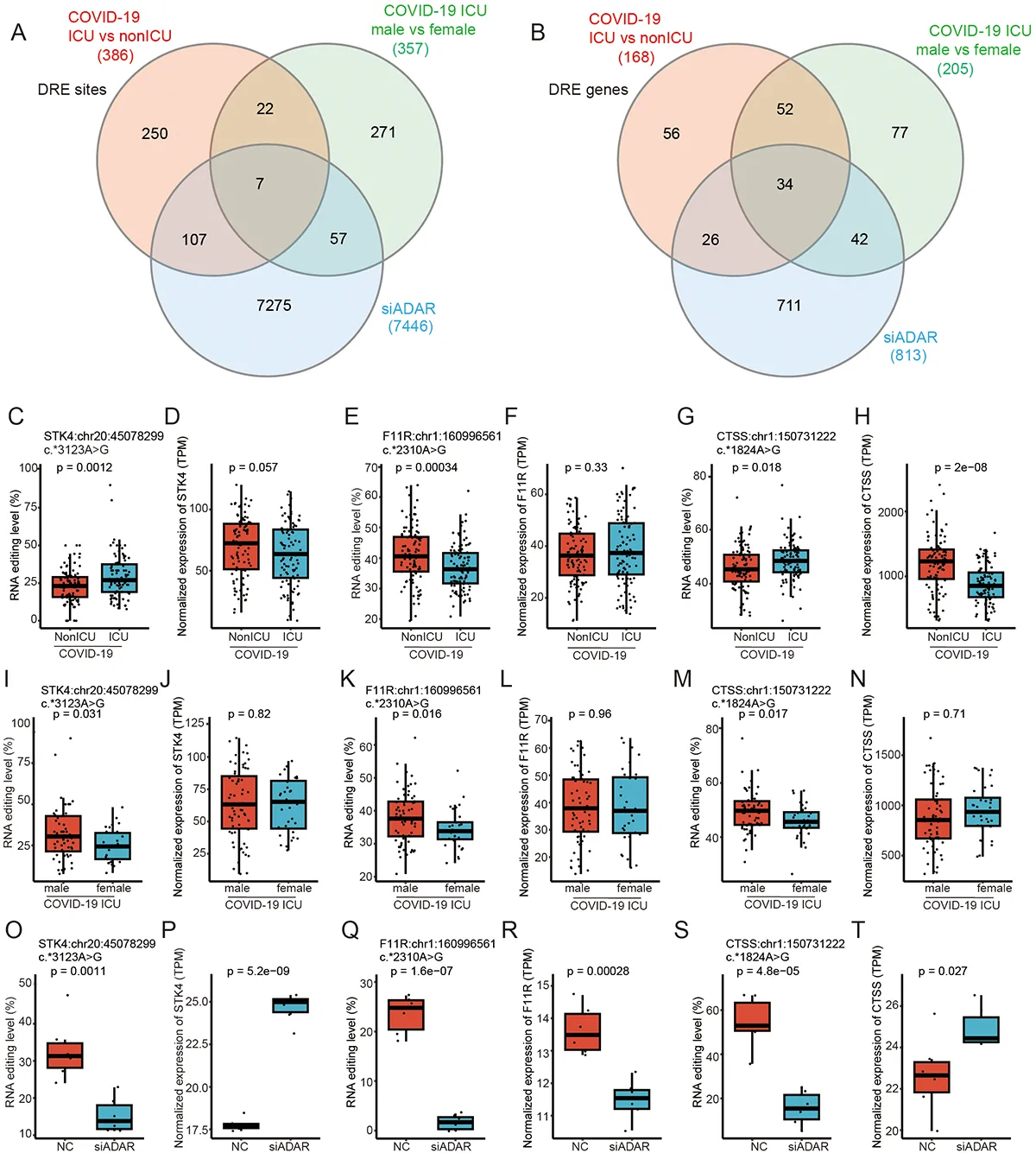
Sex-specific core RNA editing sites coordinate antiviral immunity and viral response in COVID-19 severity. (A-B) The Venn diagram shows the shared DRE sites (A) and genes (B) across COVID-19 severity, COVID-19 sex, and si*ADAR*. (C-H) *STK4*:chr20:45078299, *F11R*:chr1:160996561, and *CTSS*:chr1:150731222 A-to-I RNA editing level (C, E, and G) and gene expression level (D, F, and H) in COVID-19 ICU and COVID-19 non-ICU, respectively (*N* = 50, respectively). (I-N) *STK4*:chr20:45078299, *F11R*:chr1:160996561, and *CTSS*:chr1:150731222 A-to-I RNA editing level (I, K, and M) and gene expression level (J, L, and N) in ICU male and ICU female, respectively (*N* = 33 for ICU male; *N* = 17 for ICU female, respectively). (O-T) in the PRJNA614963 dataset, *STK4*:chr20:45078299, *F11R*:chr1:160996561, and *CTSS*:chr1:150731222 A-to-I RNA editing level (O, Q, and S) and gene expression level (P, R, and T) in A549 cells treatment with si*ADAR* and NC, respectively (*N* = 3 for NC; *N* = 3 for si*ADAR*, respectively). *p*-values are calculated using the independent *Student’s* t-test. All tests were two-sided, and a *p*-value < 0.05 was considered statistically significant.

### Genes with sex-associated RNA editing tended to be multi-edited in severe COVID-19

Our findings highlight the critical role of genes with multiple RNA editing sites in COVID-19 ICU males and COVID-19 ICU females. A total of 115 genes, representing 94% of the DRE genes, were identified to contain two or more editing sites in COVID-19 ICU males, as listed in Table S3. A total of 135 genes, representing 93% of the DRE genes, were found to contain two or more editing sites in COVID-19 ICU females, as listed in Table S4.

### Functional validation of ADAR-mediated *cis*-regulatory editing in the *CTSS* 3*’* UTR

*Sanger* sequencing was used to validate the presence of A-to-I RNA editing events at the target sites. The knockdown efficiency of si*ADAR* was validated by quantitative real-time PCR (qRT-PCR). Compared with the negative control (NC), transfection with si*ADAR* significantly reduced *ADAR* mRNA expression (Figure 6(A)). The results showed that the *CTSS*:chr1:150731222 variants were detected at the RNA level but not at the DNA level, and that editing levels were lower in si*ADAR* than in NC (Figure 6(B,C)). RNA stability assays showed that *CTSS* degraded more slowly than the control group after actinomycin D treatment with siADAR (Figure 6(D)). Consistently, knockdown of *ADAR* significantly decreased editing levels at *APOBEC3C*:chr22:39018907 in comparison with NC (Figure 6(E)).

**Figure 6.**
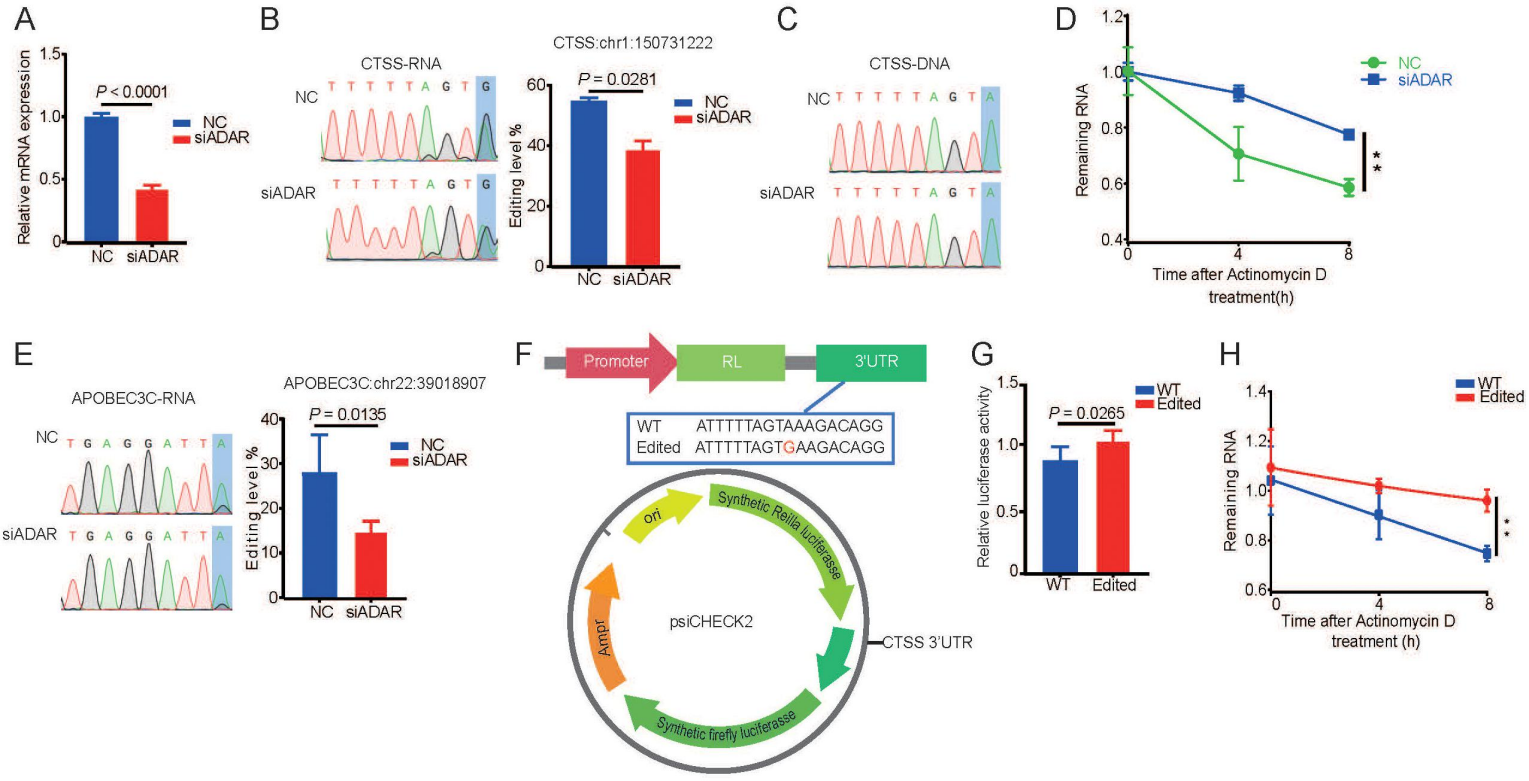
Validation of *ADAR*-dependent editing and its cis-regulatory role at the *CTSS* locus. (A) Validation of si *ADAR*-mediated knockdown efficiency. (B-C) *Sanger* sequencing peaks for *CTSS*: chr1: 150731222 at the RNA (B) and DNA (C) levels. (D) Time-course analysis of endogenous *CTSS* mRNA stability treated with siADAR after actinomycin D treatment, measured as relative mRNA expression over time (hours). (E)*Sanger*sequencing peaks for*APOBEC3C*: chr22: 39018907. (F) Schematic of luciferase reporter constructs containing wild-type (wt) or edited 3′ UTRs. (G) Relative luciferase activity for wt and edited constructs. (H) Time-course analysis of wt and edited mRNA stability after actinomycin D treatment, measured as relative mRNA expression over time (hours).*p*-values are calculated using the independent*Student’s* t-test. All tests were two-sided, and a *p*-value < 0.05 was considered statistically significant. **, *p* < 0.01.

To investigate the functional consequences of ADAR-mediated editing at the *CTSS*:chr1:150731222, we generated wild-type (WT) and edited reporter constructs, with the latter harbouring an A-to-G substitution at the edited adenosine of the editing site (Figure 6(F)). In functional reporter assays, the edited 3’ UTR of *CTSS* significantly activated the luciferase activity relative to the WT (Figure 6(G)). Notably, RNA stability assays demonstrated that the edited 3’ UTR exhibited slower degradation compared to the WT following actinomycin D treatment (Figure 6(H)).

### RBP binding site prediction of *CTSS*:chr1:150731222

To explore whether the editing site within the *CTSS* 3’ UTR lies in a region relevant to post‑transcriptional regulation, we performed in silico prediction of RNA‑binding protein (RBP) binding motifs flanking this site. The analysis identified 66 predicted RBP binding sites in total. Among these, five sites were unique to the wild‑type transcript, four sites were unique to the edited form, and the remaining 57 sites were predicted on both versions (Figure S3A). The proteins uniquely associated with the wild‑type sequence included HNRNPDL, PRR3, QKI, RBMS3, and SF1, whereas the edited transcript showed unique predictions for EIF4G2, HNRNPF, RBM28, and SRSF8 (Figure S3A). The most frequent RBPs potentially interacting with the CTSS:chr1:150731222 editing site were RBMS3, HNRNPDL, QKI, etc. (Figure S3B).

## Discussion

Prior to this study, the epitranscriptomic landscape of A-to-I RNA editing in peripheral blood and its contribution to the clinical heterogeneity of COVID-19, particularly the pronounced male bias in severe outcomes, remained largely unexplored. Here, through editome profiling of 126 blood samples from non-COVID controls and patients across mild to critical illness, we identified 1,466 differentially edited sites in 514 genes associated with disease severity. Seven core sites, with Cathepsin S (*CTSS*) at the forefront, showed robust associations with both disease severity (Figure 5(G,H)) and sex-biased regulation in critically ill patients (Figure 5(M,N)). Mechanistically, *ADAR* was elevated in the circulation of SARS-CoV-2-infected individuals relative to healthy controls but was markedly downregulated in ICU patients – a complete reversal that was significantly more pronounced in males (Figures 3(A); 4(A)); this reduction correlated with lower global editing activity, altered expression of edited transcripts, and individual differential editing events (Figure 4(C,D,H)). Collectively, these data delineate a severity-dependent dysregulation of ADAR/ADARB1 that remodels the blood epitranscriptome, potentially dampening antiviral immunity and contributing to male-biased severity in COVID-19.

The consistently stronger correlations observed with ADAR than with ADARB1 across multiple independent comparisons (Figures 2(E,F); 3(F,G); and 4(C,D)) point to a more prominent functional involvement of ADAR in the context of our study. This differential pattern is biologically plausible: ADAR is ubiquitously expressed and plays a critical role in suppressing innate immune activation by editing endogenous double‑stranded RNAs to prevent their recognition by cytosolic RNA sensors [42]. In contrast, ADARB1 exhibits a more restricted expression pattern, with its primary physiological functions centred on the nervous system [43]. The association between editing activity and clinical severity thus appears to be predominantly driven by ADAR, underscoring its central role in immune modulation during viral infection.

This self-perpetuating circuit, whereby hyperinflammation may suppress ADAR-mediated self-tolerance, thereby potentiating antiviral signalling, could constitute one mechanism underlying the male-predominant hyperinflammatory pathology of severe COVID-19 [44].

A critical observation of our study is that sex-based differences in ADAR expression and RNA editing profiles were not detectable when COVID-19 patients were analysed as a homogeneous group, but emerged exclusively within the ICU cohort. This pattern – absence of baseline disparity with amplification under physiological stress – is consistent with an emerging paradigm in immunology: sex differences in immune responses are often context-dependent and become most apparent during intense inflammatory challenge [45]. Several non-mutually exclusive mechanisms could explain this phenomenon. First, oestrogen has been shown to upregulate *ADAR* expression through oestrogen response elements in the *ADAR* promoter region [46], which may provide females with a protective buffer against *ADAR* downregulation during the hyperinflammatory state of critical illness. Second, X-chromosome dosage effects – including escape from X-inactivation of immune-related genes on the X chromosome – may confer differential regulatory capacity to females under stress conditions [47]. Third, the magnitude of the inflammatory response itself may differ between sexes: males tend to exhibit higher levels of pro-inflammatory cytokines (e.g. IL-6, TNF-α) during severe infection [48], and this more intense inflammatory milieu could drive a stronger negative feedback suppression of *ADAR* expression. Regardless of the precise mechanism, our data demonstrate that the dysregulation of A-to-I editing constitutes a molecular signature that distinguishes male from female trajectories specifically under the physiological stress of critical illness, rather than reflecting pre-existing baseline disparities in SARS-CoV-2 susceptibility.

The pooled and stratified analyses each contributed distinct information for interpreting *ADAR* and *ADARB1* expression in relation to ICU admission and sex, and together they provided a more comprehensive view of the expression patterns. We began with a global characterization of the eight clinical subgroups (Figure 1(F–H)), which revealed that *ADAR* expression varied significantly across both sex and severity strata, with opposing trends observed between males and females. This observation prompted us to disaggregate the data stepwise. We first stratified by infection status to compare COVID‑19 patients with non‑COVID controls (Figure 2(A–D)). Among infected individuals, we further examined the effects of ICU admission (Figure 3(A–E)) and sex (Figure S2A–D) separately. Notably, sex‑associated differences were only evident within the ICU subgroup, leading us to focus specifically on sex comparisons among critically ill patients (Figure 4(A,B)). This sequential approach-from global overview to progressively refined stratification-enabled us to pinpoint the context in which sex‑dependent expression differences emerge, rather than assuming a uniform sex effect across all clinical conditions.

Male sex is a well-established risk factor for adverse clinical outcomes in COVID-19. However, a critical and underexplored distinction remains whether this disparity reflects a higher inherent susceptibility to SARS-CoV-2 infection or a conditional exacerbation of disease severity once infection has occurred. Epidemiological data indicate that the risk of SARS-CoV-2 acquisition is comparable between males and females, whereas the excess morbidity and mortality observed in males manifests predominantly among critically ill patients [49]. In accordance with this framework, our sex-stratified analysis of COVID-19 patients without regard to disease severity detected no significant sex-based differences in either *ADAR* expression or global RNA editing profiles (Figure S2A-S2D), effectively ruling out pre-existing baseline disparities and the notion that SARS-CoV-2 infection per se generates sex-biased editing responses. Notably, the pronounced downregulation of *ADAR* and *ADARB1*, along with the differential RNA editing events identified in our study, emerged exclusively within the ICU cohort and was disproportionately amplified in males. This pattern delineates a severity-dependent, rather than infection-intrinsic, sexual dimorphism, wherein the dysregulation of A-to-I editing constitutes a molecular signature that distinguishes male from female trajectories specifically under the physiological stress of critical illness. By linking a well-documented clinical observation to a concrete epitranscriptomic perturbation, our findings provide a mechanistic basis for moving beyond descriptive epidemiology to interrogate the regulatory architecture that shapes male-biased disease severity in COVID-19 [49].

Among the seven core differentially edited sites identified in this study, the 3’ UTR editing event in *CTSS* emerged as the most prominent candidate bridging disease severity with sex-specific epitranscriptomic reprogramming. *CTSS* encodes a lysosomal cysteine protease that occupies a non-redundant position in adaptive immunity: it is the principal enzyme responsible for degrading the invariant chain chaperone of MHC class II molecules, a proteolytic step that is rate-limiting for antigen loading and subsequent CD4+ T cell activation [50,51]. Beyond its canonical role in professional antigen-presenting cells, *CTSS* is also expressed in endothelial compartments [52], where it contributes to extracellular matrix remodelling and amplifies local inflammatory circuits [53]. Notably, existing functional annotations have linked *CTSS* to coronavirus biology and antiviral immune activity, further highlighting its relevance to SARS-CoV-2 pathogenesis [54]. Multiple lines of evidence implicate *CTSS* in COVID-19 pathophysiology. Proteomic profiling of sera from infected patients has revealed that circulating *CTSS* levels correlate with disease severity [55]. At the molecular level, the SARS-CoV-2 spike protein is susceptible to proteolytic processing by cathepsin S, facilitating viral entry, and genome-wide CRISPR screens have independently identified *CTSS* as a host dependency factor required for efficient infection [56,57]. These findings collectively establish *CTSS* as a functional node linking viral entry, host protease activity, and clinical deterioration in COVID-19.

Prior work established *CTSS* as a direct ADAR editing target: a conserved stem-loop structure formed by inverted Alu elements within the *CTSS* 3’ UTR is recognized and edited by ADAR, which enables recruitment of the RNA-stabilizing protein HuR, thereby enhancing *CTSS* mRNA stability [58]. This editing-dependent regulatory axis is triggered by hypoxia and inflammatory cytokines, linking epitranscriptomic control to the hypoxic-inflammatory environment typical of severe COVID-19 [59,60]. In our results, *CTSS* ranked first among the seven core DRE sites, with editing levels significantly elevated in ICU patients and showing a disproportionately greater divergence between males and females. Notably, sex-specific *CTSS* regulation was functionally demonstrated in an independent immunological setting: in a murine lupus model, dendritic cell-specific Prdm1 deficiency led to selective *CTSS* upregulation and altered MHC-II-restricted antigen presentation exclusively in female mice, and pharmacological *CTSS* inhibition reversed lupus phenotypes only in females [61], indicating that *CTSS*-mediated immune pathways can operate in a female-specific manner. The convergence of these findings with our observations is striking: the *CTSS* editing event identified here resides within the 3’ UTR, positioning it to modulate transcript stability, translational efficiency, or subcellular localization [62–64]. We therefore propose that *ADAR/ADARB1* downregulation in severe COVID-19 - a decline disproportionately amplified in males – may disrupt the editing landscape of *CTSS*, potentially affecting antigen processing kinetics, skewing CD4+ T cell help, and contributing to the divergent immune trajectories observed between sexes [65]. This mechanistic framework is further reinforced by the independent identification of *CTSS* as a differentially edited gene associated with both severity and complications in paediatric Mycoplasma pneumoniae pneumonia [66,67], suggesting that *CTSS* editing may constitute a broadly conserved epitranscriptomic checkpoint across distinct respiratory pathogens. Collectively, the dual annotation of *CTSS* as an RNA editing substrate and a coronavirus-relevant immune effector, together with its experimentally validated sex-biased regulation, positions it as a compelling molecular hub through which A-to-I editing orchestrates sex-specific immune responses in critical COVID-19.

Our study suggests a sex‑dependent dysregulation of A‑to‑I RNA editing in COVID‑19, with ADAR‑mediated editing increased in mild cases but markedly reduced in ICU patients, especially males. The editing event at the *CTSS* 3’ UTR, linked to enhanced mRNA stability, suggests a potential post‑transcriptional route that may fine‑tune immune responses. However, it must be emphasized that these findings, derived from clinical cohort data, reflect correlations rather than causality, and whether the observed editing changes are drivers of severity, consequences of host response, or mere epiphenomena remains unclear. Nevertheless, these findings collectively point to an association between editing dysregulation and disease severity, offering a plausible molecular clue for the male predisposition, and the robust patterns are hypothesis‑generating. Therefore, this study should be viewed as exploratory, and future mechanistic investigations-such as siRNA‑mediated knockdown of *ADAR* in relevant cell types followed by LPS stimulation, along with functional editing assays and animal models-are needed to unravel the biological relevance of ADAR‑mediated RNA editing in SARS‑CoV‑2 infection and its potential contribution to sex disparities in clinical outcomes.

Additional genes at the intersection of DRE events and ADAR perturbation, *STK4* and *F11R*, may further link altered A-to-I editing to immune and vascular dysfunction in severe COVID-19. *STK4* encodes the kinase MST1, a regulator of Hippo signalling that also controls antiviral innate immunity [68]; its deficiency impairs type I, II, and III interferon responses by disrupting TBK1-IRF3 signalling [69], and both *STK4* mRNA and protein levels are significantly reduced in COVID-19 patients relative to healthy controls. *F11R* encodes Junctional Adhesion Molecule-A (JAM-A), an endothelial and platelet adhesion receptor that also serves as a viral entry receptor for reovirus and rotavirus and mediates leukocyte transendothelial migration [70,71]. Although direct evidence linking either gene to severity-dependent or sex-specific editing in COVID-19 is currently lacking, their established roles in interferon-mediated antiviral defence and vascular barrier regulation [72,73], respectively, align with both our editing data and the hallmarks of severe COVID-19, underscoring their potential relevance to the epitranscriptomic dysregulation that characterizes critical illness [74].

In summary, our integrated study delineates a severity-dependent landscape of A-to-I RNA editing in COVID-19 that becomes sex-biased specifically during critical illness. The downregulation of *ADAR/ADARB1* in ICU patients, which was disproportionately pronounced in males, coupled with the identification of disease-severity-associated editing events in immune-related genes, highlights the pivotal role of the epitranscriptome in shaping disease heterogeneity. Functional validation of the ADAR-*CTSS* regulatory axis provides a mechanistic foundation for these observations. These DRE sites represent promising candidates for biomarker-based patient stratification. Future research is warranted to clarify how specific editing events affect protein function and their direct role in disease development, which could help in developing new therapeutic approaches.

## Supplementary Material

Supplemental Material

Fig S2.jpg

Fig S3.jpg

Supple Tables.xlsx

Fig S1.jpg

## Data Availability

The study’s key findings are comprehensively outlined in the article, and any additional questions may be forwarded to the corresponding authors for clarification.
