# Supplementary material for: Severity-dependent and sex-biased dysregulation of A-to-I RNA editing in COVID-19 reveals CTSS as a key regulatory target
Source: RNA Biol. 2026 Oct 14;23(1):1–20. doi: 10.1080/15476286.2026.2736869 (PMC13671454; doi:10.1080/15476286.2026.2736869)
Supplement: Supplementary file 1 [file KRNB_A_2736869_SM8428.docx]

Figure Legends

Figure S1. The three 3′ UTR DRE sites with *cis*-regulatory effects in COVID-19 and non-COVID-19. (A-F) *APOBEC3C*:chr22:39018907, *MDM2*:chr12:68843778, and *FCGR3A*:chr1:161542063 A-to-I RNA editing level (A, B, and C) and gene expression level (D, E, and F) in non-COVID-19 and COVID-19 (N = 100 for COVID-19; N = 26 for non-COVID-19, respectively). (G-I) The *Spearman* correlation of *APOBEC3C*:chr22:39018907 (G), *MDM2*:chr12:68843778 (H), and *FCGR3A*:chr1:161542063 (I) editing level and gene expression level in two groups. *P*-values are calculated using the independent *Student's* t-test. All tests were two-sided, and a *p*-value < 0.05 was considered statistically significant.

Figure S2. A-to-I RNA editing in COVID-9 females and COVID-19 males. (A) The mRNA expression of *ADAR*. (B-D) The number of edited sites (B), genes (C), and editing level (D) between females and males in COVID-19 (N = 38 for COVID-19 females; N = 62 for COVID-19 males, respectively). *P*-values are calculated using the independent *Student's* t-test. All tests were two-sided, and a *p*-value < 0.05 was considered statistically significant.

Figure S3. RBP binding site prediction of CTSS:chr1:150731222. (A) The Venn plot of RBP-binding sites on CTSS-3' UTR WT and edited sites. (B) The most frequent RBPs that potentially interact with the CTSS:chr1:150731222 editing site.

Table S1. The primers for PCR amplification, *Sanger* sequencing, and qPCR, as well as the sequences for scramble siRNA and ADAR siRNA.

Table S2. The DRE sites between COVID-19 NonICU and COVID-19 ICU groups.

Table S3. Enrichment of differential A-to-I RNA editing genes with multiple editing sites in males.

Table S4. Enrichment of differential A-to-I RNA editing genes with multiple editing sites in females.
